# Exploring allosteric communication in multiple states of the bacterial ribosome using residue network analysis

**DOI:** 10.3906/biy-1802-77

**Published:** 2018-10-25

**Authors:** Özge KÜRKÇÜOĞLU

**Affiliations:** 1 Department of Chemical Engineering, Faculty of Chemical-Metallurgical Engineering, İstanbul Technical University , İstanbul , Turkey

**Keywords:** Allosteric communication, closeness centrality, betweenness centrality, drug-resistant bacteria, druggable sites

## Abstract

Antibiotic resistance is one of the most important problems of our era and hence the discovery of new effective therapeutics is urgent. At this point, studying the allosteric communication pathways in the bacterial ribosome and revealing allosteric sites/residues is critical for designing new inhibitors or repurposing readily approved drugs for this enormous machine. To shed light onto molecular details of the allosteric mechanisms, here we construct residue networks of the bacterial ribosomal complex at four different states of translation by using an effective description of the intermolecular interactions. Centrality analysis of these networks highlights the functional roles of structural components and critical residues on the ribosomal complex. High betweenness scores reveal pathways of residues connecting numerous sites on the structure. Interestingly, these pathways assemble highly conserved residues, drug binding sites, and known allosterically linked regions on the same structure. This study proposes a new residue-level model to test how distant sites on the molecular machine may be linked through hub residues that are critically located on the contact topology to inherently form communication pathways. Findings also indicate intersubunit bridges B1b, B3, B5, B7, and B8 as critical targets to design novel antibiotics.

## 1. Introduction

**Figure 1:**
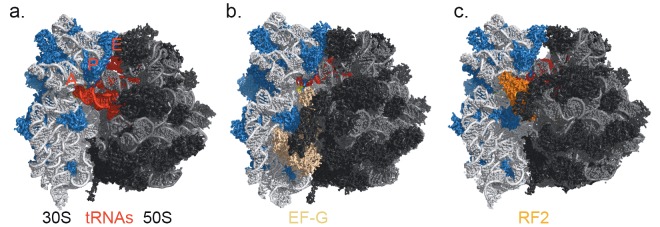
Bacterial ribosome complex with (a) mRNA and A-, P-, and E-tRNAs (in red); (b) EF-G (in wheat) and hybrid state P/E-tRNA; (c) RF2 (in orange) and P- and E-tRNAs. Ribosomal proteins on the small subunit 30S are shown in blue and on the large subunit 50S in black.

The ribosome is the molecular machine that synthesizes proteins in the cell across all kingdoms of life. While the size of the complex changes from one organism to another, its molecular structure is mainly maintained by two differently sized subunits. The small subunit, called 30S in bacteria, accommodates mRNA carrying the genetic code from the nucleus to be translated on 30S in the decoding center (DC). This small site on the supramolecule is formed of highly conserved residues G530, A1492, and A1493 (throughout this study, the numbering scheme of Thermus thermophilus is used unless stated otherwise) (Moazed and Noller, 1990; Yoshizawa et al., 1999; Demeshkina et al., 2012) . Acylated-tRNAs are delivered by the elongation factor-Tu (EF-Tu).GTP complex, which docks the 70S near the A-site. After the correct codon-anticodon pairing, GTP hydrolysis releases EF-Tu from the complex, leaving the acetylated-tRNA behind (Schmeing et al., 2009) . Temporary accommodation of the tRNA starts from the A/T site of the complex, which is respectively translocated to A-, P-, and E-sites (Figure 1a) (Agrawal et al., 1996) . The translocation of tRNAs and mRNA to the next binding sites is catalyzed by EF-G docking at the subunit interface (Figure 1b) (Agrawal et al., 1998) . The peptide bond synthesis between two amino acids attached to the CCA ends of tRNAs at the A- and P-sites is catalyzed on the large subunit, called 50S in bacteria, at the peptidyl transferase center (PTC). This site is formed of only RNAs, which are highly conserved. The growing polypeptide chain exits the complex from the ribosomal tunnel, which is ~100 Å long (Nissen et al., 2000). The stability of the complex is maintained by many intersubunit bridges involving RNA–RNA, RNA–protein, and protein–protein contacts between 30S and 50S (Liu and Fredrick, 2016). At the end of the elongation phase, stop codons on the mRNA are recognized by release factors (RFs) RF1 and RF2 (Korostelev et al., 2008) (Figure 1c) and then the subunits dissociate.

Correct translation of the genetic code to a functional protein is a vital process for the cell. Therefore, the 2.5-MDa complex ensures this by employing highly sophisticated mechanisms, such as allostery (Karbstein, 2013). Many mutational and structural studies have revealed that distant regions of the ribosome complex communicate with each other by using conformational changes and tertiary interactions (Polacek and Mankin, 2005; Chan et al., 2006; Sothiselvam et al., 2014). For example, DC and PTC are allosterically linked as shown by extensive studies on yeast (Rakauskaitė and Dinman, 2008; Rhodin and Dinman, 2011) and bacteria (Laurberg et al., 2008). Long-distance communication between DC and the EF-Tu implies that the GTPase activity of EF-Tu may be enhanced by allostery as well (Schmeing et al., 2009; Meskauskas and Dinman, 2010; Adamczyk and Warshel, 2011) . On the other hand, various studies indicate that PTC is another region constantly communicating with other sites, such as with EFs (Chan et al., 2006; Rakauskaitė and Dinman, 2008; Meskauskas and Dinman, 2010) and the ribosomal tunnel (Vazquez-Laslop et al., 2008; Ramu et al., 2011) .

While these experimental studies reveal which sites are cooperating in an allosteric communication, the mechanism of how a perturbation on one site propagates to another still remains elusive. At this point, protein contact topology and residue network models offer a key to understand these molecular mechanisms by highlighting the important structural features of the macromolecule (Csermely et al., 2013; Di Paola and Giuliani, 2015), and to identify allosteric pathways between functional regions of the proteins (Yan et al., 2014; Di Paola and Giuliani, 2015; Amor et al., 2016; Dokholyan, 2016) .

Motivated by the success of these studies, we recently examined the ribosome structure from this perspective and determined potential allosteric communication pathways between the DC and PTC, and between the ribosomal tunnel and PTC (Guzel and Kurkcuoglu, 2017). Our analysis based on contact topology successfully pinned numerous known allosteric and drug binding sites on the calculated shortest pathways, which were altered according to translation state during protein synthesis. In this study, we further examine the ribosome complex structure topology in terms of centrality measures to reveal hub residues that may be important in the allosteric mechanism of the ribosome. Previously, residue network analysis has been employed to characterize RNA structure (Lescoute and Westhof, 2006), to study the conformational space of tRNA (Wuchty, 2003) , and to understand the nature and coevolutionary patterns of amino acid–nucleotide contacts in the ribosome (Mallik et al., 2015; Mallik and Kundu, 2017). In addition, degree, closeness, and betweenness measures were studied for the ribosomal subunits and intact ribosomal complexes from different organisms, while keeping focus on closeness and evolutionary conservation of DC and PTC residues (David-Eden and Mandel-Gutfreund, 2008).

Effective translation of the genetic code into a functional protein is clearly ensured by sophisticated mechanisms coordinated by key residues, which become target points especially for bacteria. A large number of antibiotics target the ribosomal complex to stop protein synthesis in bacteria (Arenz and Wilson, 2016). However, while bacteria gain resistance to conventional antibiotics, studies focus on revealing intriguing mechanisms employed by the ribosome and exploring weak points, i.e. key residues, of the bacterial ribosome. Here, different from previous studies on residue networks of the ribosome, (i) four distinct translation states of the intact ribosomal complexes for the same organism, Thermus thermophilus , are investigated; (ii) three centrality measures of various structural components are compared; (iii) emphasis is given to the betweenness centrality, which indicates potential allosteric pathways based on the topology; and (iv) new druggable sites are proposed.

## 2. Materials and methods

### 2.1. Dataset

Four different ribosomal complex structures of Thermus thermophilus outlining the protein synthesis investigated in this study. Three of these crystal structures are aij = N ij N i .N j (1) belong to the elongation phase, with PDB IDs 2j00-2j01 (Selmer et al., 2006) , 4juw-4jux (Tourigny et al., 2013) , and 2wdk-2wdl (Voorhees et al., 2009) , and one to the translation termination phase with PDB ID 3f1e-3f1f (Korostelev et al., 2008). The ribosome complex at its pretranslocational state (2j00-2j01) includes mRNA and some portion of A-, P-, and E-tRNAs; the intermediate state of the translocation structure (4juw-4jux) accommodates mRNA, P/E-tRNA, and EF-G.GDP; the prepeptidyl transfer state structure (2wdk-2wdl) contains mRNA and A-, P-, and E-tRNAs as shown in Figure 1. The translation termination state structure (3f1e-3f1f) includes mRNA, P-tRNA, E-tRNA, and RF2. Ribosomal complexes contain ~11000 residues and resolutions of all structures are under 3.0 Å. Helices on the small subunit will be referred to with ‘h’ whereas those on the large subunit are referred to with ‘H’.

### 2.2. Graph model

The ribosome structure is described as a weighted bidirectional graph formed of nodes and edges. Nodes are placed at the alpha-carbon of amino acids and the phosphorus atoms of nucleotides. Neighboring nodes are linked by edges. The length of edges is calculated based on the local interaction strength $a_{\mathrm{ij}}$ of the residues defined as:

$a_{\mathrm{ij}}=\frac{N_{j}}{\sqrt{N_{i}N_{j}}}$

**Figure 2:**
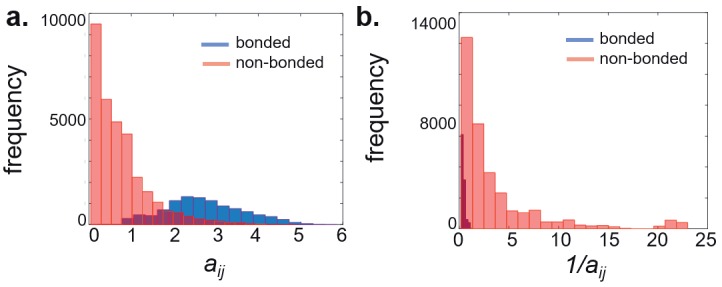
Distributions of (a) local interaction strengths and (b) edge lengths.

Here, N_ij_ is the total number of atom–atom contacts of the ith and jth residues falling within a cutoff distance of 4.5 Å, where van der Waals and electrostatic interactions occur. This cutoff value is commonly used to determine atom– atom interactions in residue network analyses (Brinda and Vishveshwara, 2005; Chennubhotla and Bahar, 2006). The Nij value is weighted by the size of the residues, i.e. Ni and Nj, to eliminate any bias due to the size of the residues in the ribosome complex. Figure 2a displays local interaction strengths for the bonded and nonbonded interactions in the ribosomal complex structure 2j00-2j01 as an example. A skewed distribution is observed for nonbonded interactions while values for the bonded interactions are normally distributed.

In the graph, short length edges imply close nodes that can share information. Therefore, the length of edges is set as 1/a_ij_ (Guzel and Kurkcuoglu, 2017), as in the relationship between potential energy and distance for electrostatic and van der Waals interactions. According to this formulation, strong bias towards covalently bonded interactions is suppressed and edges describing both covalent and longrange interactions important for the topology are included in the calculations (Figure 2b).

In order to characterize the protein topology network, centrality is a good measure for identifying the importance and topological roles of nodes in the network. The most useful centrality measures are degree, closeness, and betweenness (David-Eden and Mandel-Gutfreund, 2008; Fokas et al., 2016). Degree centrality is the total number of edges linked to a node. Closeness centrality CC(l) is defined as the inverse of the average shortest path length d_lj_ between residue l and the other nodes by:

$C_{C}(l)=\frac{N}{\sum_{j}^{}d_{\mathrm{ij}}}$ (2)

Betweenness of a node l, CB(l), is a useful measure to explore highly connected nodes indispensable in linking distant sites of the network.

$C_{B}(l)=\sum_{i\sigma j\sigma i}^{}\frac{\sigma_{\mathrm{ij}}(l)}{\sigma_{\mathrm{ij}}}$ (3)

Here, σ_ij_ is the number of shortest paths between the ith and jth nodes and σ_ij_(l) is the number of shortest pathways linking i and j, passing through node l. High values of CB point to hubs of the graph.

In this study, centrality measures of all four ribosome structures are calculated and discussed using the graph theory approach. As a case study, the ribosome complex topology at the translocation step, i.e. 4juw-4jux, is further investigated to reveal hub residues of the formed graph, which successfully indicates functionally critical highly conserved regions.

The residue networks are constructed with an inhouse code written in Fortran77 and centrality analysis is performed using an in-house code in MATLAB with the academic license of İstanbul Technical University.

## 3. Results

### 3.1. Centrality analysis of intact ribosome structures

**Figure 3:**
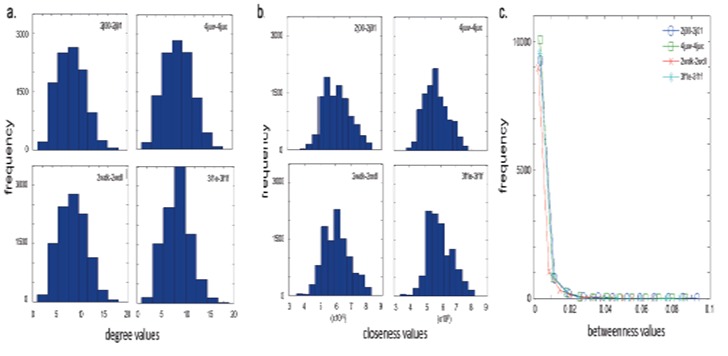
Probability density function of (a) degree, (b) closeness, and (c) betweenness scores.

The measure of degree reflects the number of neighbors of each amino acid and nucleotide within a cutoff distance of 4.5 Å. This distance includes nonbonded interactions that can transmit a perturbation on an allosteric site to the distant functional region by conformational rearrangements. Similar to a previous study (David-Eden and Mandel-Gutfreund, 2008), degrees for residues in all structures display a normal distribution with a mean of ~8.5 ± 2.7 when considering the intact structure of the complex (Figure 3a). The ribosome complex is formed of RNA and proteins; therefore, residue specificity should be considered in the centrality analysis. Amino acids make more interactions compared to nucleotides of rRNA and tRNAs, while tRNAs have the least number of neighbors (Table 1).

**Table 1:** Mean values of centralities in different structural components.

PDB ID	Degree			Closeness (×10^–6^)		
	Amino acid	rRNA	tRNA	Amino acid	rRNA	tRNA
2j00-2j01	9.0 ± 2.7	7.2 ± 2.5	5.6 ± 1.7	5.7 ± 0.7	6.3 ± 0.8	7.2 ± 0.5
4juw-4jux	9.4 ± 2.7	7.6 ± 2.5	6.2 ± 1.9	5.4 ± 0.6	6.0 ± 0.7	6.7 ± 0.3
2wdk-2wdl	9.2 ± 2.6	7.5 ± 2.5	5.6 ± 1.8	5.7 ± 0.7	6.4 ± 0.8	7.1 ± 0.4
3f1e-3f1f	9.3 ± 2.6	7.4 ± 2.5	5.9 ± 1.8	5.7 ± 0.7	6.2 ± 0.8	7.0 ± 0.6

**Figure 4:**
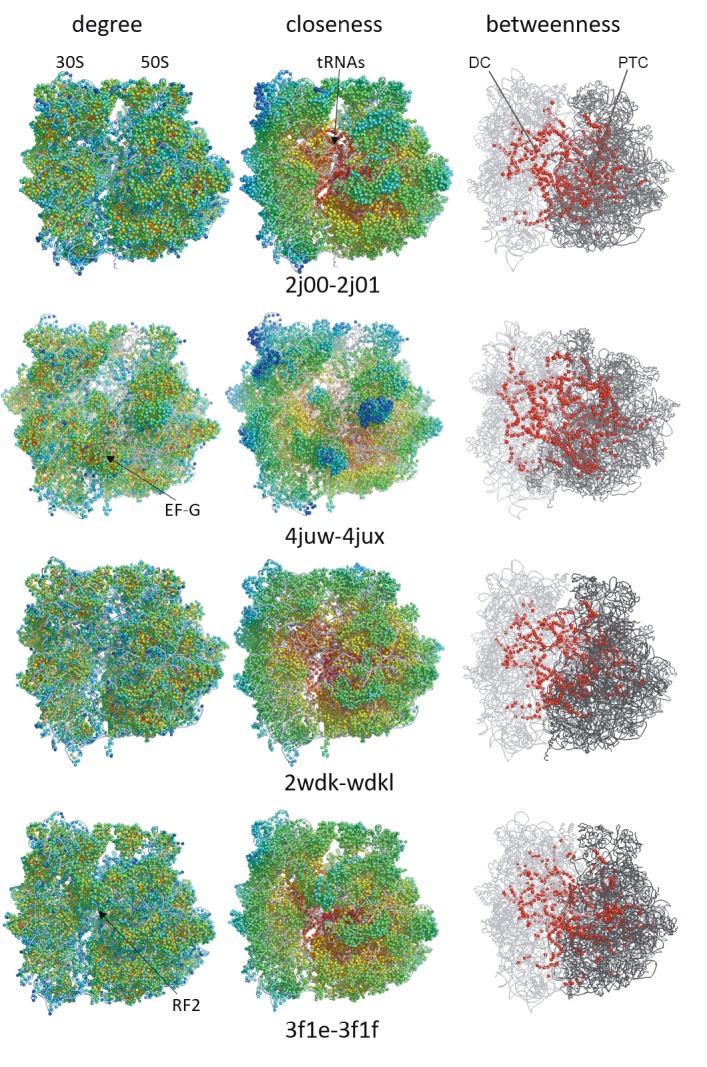
Degree and closeness scores are colored from blue (lowest) to red (highest), respectively, in the left and middle panels. In the right panel, betweenness scores of residues above the 0.95 quantile are colored red on 30S (light gray) and 50S (dark gray).

We also analyzed closeness centrality for the ribosomal topology. Closeness measures the extent of interactions of a residue, either directly with others or through few neighbors. Different translation conformations of the intact complex exhibit distributions with similar mean values of ~6 × 10–6 and standard deviation of ~0.7 × 10–6 (Figure 3b). Residues with high closeness values are clustered at the interface of the complex, encompassing highly conserved regions, namely DC, PTC, h44, and H69, as well as tRNAs, all composed only by RNA. Here, the long helix h44 spanning from the neck to the spur region of the small subunit comprises four intersubunit bridges, namely B2a, B3, B5, and B6 (Liu and Fredrick, 2016). Closeness measures are further investigated focusing on different structural components of the molecular machine, i.e. proteins, rRNAs, and tRNAs (Table 1). The main structure of the ribosome is formed of rRNAs. Ribosomal proteins are usually located on the rRNA surface, to which they are anchored by long polymeric extensions penetrating to the core of the complex. Due to this structural layout, amino acid residues exhibit low closeness values compared to rRNAs (Table 1; Figure 4). On the other hand, the ribosomal structure assumes the highest closeness scores for tRNAs, which have the lowest degree values.

Betweenness values for the ribosome residue networks are calculated for the dataset. By definition, nodes (residues) with high betweenness centrality mediate the flow of information between distant sites and their absence/mutation would disconnect the communication of these regions (David-Eden and Mandel-Gutfreund, 2008; Koschützki and Schreiber, 2008; Fokas et al., 2016). Figure 3c displays frequency distributions of betweenness scores following power law. Accordingly, a small percentage of residues, ~5%, are at the top 0.05 quantile (>0.013), indicating that they take part in numerous shortest pathways spanning the network. These residues are distinguished as hubs linking distant sites of the ribosomal structure. When the top 0.05 quantile is mapped on the native structures, tRNAs, intersubunit bridges, proteins EF-G and RF2, and many drug binding sites are highlighted (Figure 4), which will be discussed later in detail. Residues with high betweenness scores are scattered differently in four structures due to the rearrangements in the contact topology. Nucleotides constitute the highest portion of the residues at the top 0.05 quantile within a range of 90%–97%, which changes according to the translation structure. Interestingly, these residues are located next to each other such that they form pathways linking distant regions of the ribosome, such as active sites DC and PTC. This observation is elaborated for the translocation complex as a case study in Section 4.3.

### 3.2. Centrality analysis of decoding center and peptidyl transferase center

**Figure 5:**
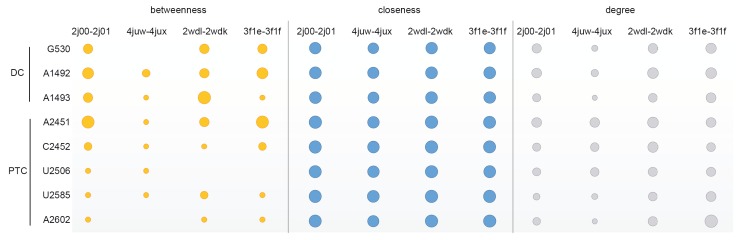
Centrality scores of residues on DC and PTC. Betweenness scores are in the range of 0.00–0.05, closeness scores are in the range of 6.2–8.3 (×10^–6^), and degree scores are in the range of 3–16.

The supramolecule ribosome has evolved in such a way that its structure determines its function: its unique shape is designed to do specific globular motions for its function, such as ratcheting of subunits for translocation, and functional residues are carefully distributed on the structure to locally ensure its cellular mission (Karbstein, 2013; Rodnina et al., 2017) . At this point, centrality analysis of the residues of DC (G530, A1492, A1493) and five critical residues at the core of catalytic site PTC (A2451, C2452, U2506, U2585, A2602) (Nissen et al., 2000; Bashan et al., 2003; Schmeing et al., 2005) is conducted to reveal network properties of the functional residues of this unique network. Figure 5 displays centrality scores of these eight nucleotides for the dataset. Previously, degree and closeness scores of residues on DC and PTC were investigated for one conformation of the Thermus thermophilus 70S complex using a similar residue network approach (Sobolev et al., 1999; David-Eden and Mandel-Gutfreund, 2008) , and they were significantly higher compared to remaining rRNA residues. In this study, we investigated four different conformations of the complex and noted that all investigated residues generally have high degree scores with respect to the mean values calculated for rRNAs (Table 1). DC nucleotides make multiple interactions in the pretranslocation (2j00-2j01), prepeptidyl (2wdk-2wdl), and termination states (3f1e3f1f). Their residue contacts seem to diminish to half in the translocation state (4juw-4jux) due to the lack of A/P-tRNA in the crystal structure. In the catalytic cavity, where local packing fluctuates according to translation state, A2451 at the A-site crevice consistently makes a high number of contacts in all structures. Interestingly, the number of interactions of the flexible U2585 and A2602 change dramatically from one conformation to the other. A2602 is crucial for peptide release in the termination state (Polacek et al., 2003) as well as in rotary motion of the CCA end of A-tRNA together with U2585 (Agmon et al., 2003) . These critical roles are clearly reflected in degree scores, increasing up to 5-fold, especially at the prepeptidyl and terminations states (from 3 to 16 for A2602).

Closeness scores of all eight residues are within the range of 6.2–8.3 × 10–6, significantly high when compared to rRNAs, as was reported before (David-Eden and Mandel-Gutfreund, 2008).

Betweenness values of all DC residues and A2451, C2452, and U2585 on PTC are at the top 0.05 quantile (>0.013), pinning these critical nucleotides at the crossroads of all shortest pathways spanning the supramolecule at all translation states studied here. U2506 and A2602 are also at the top 0.05 quantile but in specific translation conformations.

## 4. Discussion

### 4.1. Contact topology–function relationship of the bacterial ribosome structure

Degree centrality analysis of the intact ribosome structures shows that the number of interactions of amino acids is higher compared to nucleotides (Table 1). This does not necessarily indicate that amino acids are more ‘important’ than nucleotides in the graph, but rather shows the difference in local packing between differently sized residues: smaller sized amino acids are more densely packed than nucleotides in the ribosome structure. tRNAs constitute the least dense region on the ribosomal complex with a small number of neighbors. This structural design enables tRNAs to adopt significant flexibility critical for their accommodation at the subunit interface during translocation (Figure 4).

In globular protein structures, residues with high closeness values usually cluster at the core of the structure, and active site residues of enzymes usually have high degree and high closeness values (Amitai et al., 2004) . The ribosome complex presents a very unique architecture: a globular shape formed of two hemispheres connected by flexible linkers. Similar to globular proteins, closeness of residues diminishes towards the periphery of the structure (Figure 4). Although tRNAs are well connected within the structure, which is reflected by high closeness, they have low degree values implying their role as flexible hinges of the residue network, as discussed for other protein structures (Fokas et al., 2016). Indeed, we previously determined that the interface of the ribosomal complex at different translation states acts like a hinge region and coordinates the low-frequency motions of two large subunits, such as in the ratchet-like rotation of the subunits (Guzel and Kurkcuoglu, 2017).

When we focus on the functional sites DC and PTC, we note that degree scores of active site residues vary for different states of the ribosomal complex (Figure 5). This observation indicates that correct interpretation of the degree centrality strongly depends on the conformational state of the protein complex. Although decoding and catalytic sites are composed of only rRNA, the ribosome does not make any exception to the general idea that active site residues have high closeness in the protein structure (del Sol et al., 2006; Fokas et al., 2016). A detailed residue network analysis of enzymatic and nonenzymatic proteins indicated that while the shapes of functional sites are different, either a cavity or a cleft, functionally important residues have high closeness centrality reflecting the topological effect of one amino acid on other residues (del Sol et al., 2006). Similarly, DC and PTC respectively hold a small area and a cavity located at the interface of the subunits with high centrality, as well as with solvent and drug accessibility (Arenz and Wilson, 2016). Betweenness scores of these functional residues fluctuate between relatively high and low values, but still in the top 0.05 quantile, according to the translation conformation. This finding supports the idea that constant communication of DC and PTC with each other as well as with the other active sites on the complex is maintained by the use of local and global conformational changes (Laurberg et al., 2008; Rakauskaitė and Dinman, 2008; Ortiz-Meoz and Green, 2010; Rhodin and Dinman, 2011; Karbstein, 2013) . Centrality calculations conducted here demonstrate that the strategical location of these small sites on the enormous complex clearly guarantees functional residues to be ‘close’ to every new piece of information with the help of their numerous contacts, i.e. to constantly monitor propagation of significant perturbations due to specific binding/unbinding events in the structure during protein synthesis.

### 4.2. Residues with high betweenness scores link small and large subunits of the bacterial ribosome

Residue network analysis in this study gives a comprehensive look at the contact topology of the ribosome complex at various translation states and highlights specific regions with high potential to take roles in allosteric communication between many distant functional sites on the molecular machine. Calculations indicate that a large number of intersubunit bridges accommodate highly connected residues supporting the modularization of the network (Figure 4), where the distinct dynamic modules are 30S and 50S, as recently reported (Guzel and Kurkcuoglu, 2017). These residues are at the top 0.05 quantile of betweenness distribution and are located on stable intersubunit bridges B2a/d and B3 and relatively more flexible bridges B1b/c, B2b, B2c, B5, B7, and B8 (Table 2). Flexible tRNAs also contain residues with high betweenness values at the subunit interface that can receive/send information between domains in the form of conformational changes.

**Table 2:** Residues on the ribosome intersubunit bridges at top 0.05 quantile of the betweenness distribution.

Intersubunit bridge	30S components	50S components	Translation state and PDB ID
B1b	Gly68 on S13	Tyr146 on L5	Translocation, 4juw-4jux
B2a/d	A1495 on h44	A1919 (C1920) on H69*	Prepeptidyl, 2wdk-2wdl
B2b	C783 on h24	C1836 on H68	Pretranslocation, 2j00-2j01
B2c	C770, G771, C899 on h27	C1832, C1833 on H67	Pretranslocation, 2j00-2j01 Prepeptidyl, 2wdk-2wdl Termination, 3f1e-3f1f
B3	A1483 on h44 A1418 on h44	C1947-G1959 on H71 G1948-C1958 on H71	Prepeptidyl, 2wdk-2wdl Translocation, 4juw-4jux Termination, 3f1e-3f1f
B5	G1475 on h44	A1689, A1700 on H62	Prepeptidyl, 2wdk-2wdl Termination, 3f1e-3f1f
B7a	A702 on h23	A1848 on H68	Termination, 3f1e-3f1f
B7b	G773 on h24	Asn202 on L2	Prepeptidyl, 2wdk-2wdl termination, 3f1e-3f1f
B8	U340 on h14	Thr96 on L14	Pretranslocation, 2j00-2j01 Translocation, 4juw-4jux Prepeptidyl, 2wdk-2wdl

*The exact intersubunit bridge residue is A1919 (Liu and Fredrick, 2016), but the predicted residue is the neighbor C1920.

Bridge B1b residues are detected as hub residues in the calculations for the translocation conformation 4juw-4jux; this dynamic bridge undergoes significant rearrangements during the swivel motion of the 30S head to translocate tRNAs (Dunkle et al., 2011). The intersubunit bridge B2a/d is predicted as a hub region at the prepeptidyl state (2wdk-2wdl), where a high number of atom–atom. contacts are established between A1495 on h44 and C1920 on H69. Residues on H69 are particularly known to adopt different conformations during tRNA selection, which is critical for recruitment of the correct acylated-tRNA to the complex (Ortiz-Meoz and Green, 2010). Next to this site, hydrogen-bonding between C783 on helix h24 and C1836 of H68 forms the bridge B2b, which is lost at the prepeptidyl transfer structure (Liu and Fredrick, 2016).

Similarly, our analysis indicates that these residues have high betweenness values at the pretranslocation state (2j00-2j01) structure. Still at the B2 region, hydrogenbonding between C1832 of H67 and C899 on h27 as well as interactions between nucleotides C770-G771 and h27 are important for the stability of the bridge B2c and subunit association (Belanger et al., 2002). These critical interactions are predicted as hubs for the pretranslocation, prepeptidyl, and termination states.

Bridge B3 is fortified by numerous hydrogenbonding interactions, since it is the pivot point during the ratcheting motion (Valle et al., 2003) . Here, two A-minor interactions are highly conserved; these are A1483 of h44 and C1947-G1959 on H71, A1418 of h44, and G1948-C1958 of H71. Network topology suggests these residues as hubs. Besides their critical role in subunit association (Pulk et al., 2006) , they have high potential in maintaining communication between two subunits at elongation and termination phases. The same long helix h44 accommodates the intersubunit bridge B5, far from the DC. Nucleotide G1475 of h44 and universally conserved A1700 on H62 interacting via hydrogen-bonding are also noted within residues with high betweenness values at the prepeptidyl and termination states.

The intersubunit bridge B7 is located at the base of the L1 stalk interacting with the E-tRNA. Bridge B7a is formed by stacking of A702 on h23 and highly conserved A1848 of H68. Near this site, bridge B7b links h23 and h24 of the small subunit and ribosomal protein L2 of the large subunit. Both sites emerge as hub regions in elongation and termination states. As bridges B7a and B7b are next to the E-site, they have high potential to rapidly transmit conformational changes on one subunit to the other. In addition, the whole E-tRNA, both in its classical and hybrid states, is highlighted to play a role in the transmission of information. Indeed, studies report the importance of E-site dynamics in maintaining translation fidelity (Nierhaus, 2006).

The B8 bridge, specifically U340 of helix h14 and ribosomal protein Thr96 on L14, also emerges as a hub region in elongation structures, according to calculations.

Disruption at this bridge formed of highly conserved residues was reported to hold the complex at the decoding stage (Schmeing et al., 2009) , suggesting the role of this area in GTPase activity of EFs. Calculations also point to B8, which seems to be part of an allosteric pathway between small and large subunits, as will be discussed in the next section.

These findings agree with our previous study on the prediction of potential allosteric pathways between DC and PTC at different states of translation, crossing intersubunit bridges B2a and B3, as well as P-tRNAs in the ribosome structures (Guzel and Kurkcuoglu, 2017). Besides giving hints about the molecular mechanisms of the ribosomal complex for correct protein translation, these results also indicate that intersubunit bridges emerge as critical regions for allostery that may be considered as drug targets.

Supporting this idea, a recent study on yeast (Gulay et al., 2017) reported the role of intersubunit bridges in providing energy transfer between two subunits during translation and in helping allosteric communication between distant sites. Moreover, allosteric control of intersubunit bridges over ribosome dynamics has been long debated, such as for B1a (Sergiev et al., 2005) , B1b/c (Rhodin and Dinman, 2011) , B2 (Wang et al., 2012) , B3 (Prokhorova et al., 2017), and B8 (Fagan et al., 2013). Moreover, mutation studies suggested that bridges B5, B6, and B8 are important parts of a network of interactions affecting decoding fidelity by changing the GTPase activity of EF-Tu (Sun et al., 2011) . There is also evidence for the role of tRNAs in allosteric communication in the ribosome structure (Schmeing et al., 2009; Sethi et al., 2009; Gregory et al., 2011) .

While maintaining stability between subunits during large conformational changes for translation, and keeping distant functional sites informed about the translation stages, two intersubunit bridges on the long h44 have another critical property in common: being drug targets (Arenz and Wilson, 2016). The antibiotic thermorubin targets the intersubunit bridge B2a linking h44 and H69, and stabilizes nucleotide C1914 in a flipout conformation sterically clashing with A-tRNA (Bulkley et al., 2012). Similarly, neomycin molecules bind to h44 and H69 near B2a and allosterically stabilize an intermediate translocation step by preventing the required conformational rearrangements in bacterial B2a (Wang et al., 2012) . In addition, capreomycin and viomycin bind near DC and B2a and stabilize a hybrid state of the bacterial ribosome complex, thus in turn preventing the translocation (Stanley et al., 2010) . A recent structural study on the eukaryotic ribosome revealed the binding site of a drug developed for a genetic disease causing infantile death near bridge B3 (Prokhorova et al., 2017).

By considering their high potential as hubs linking functional sites at the subunit interface, bridges B1b, B3, B5, B7, and B8 are attractive regions for novel drug design to kill malign bacteria, which rapidly gain resistance to conventional antibiotics.

### 4.3. Potential allosteric pathways on the translocation complex

**Figure 6:**
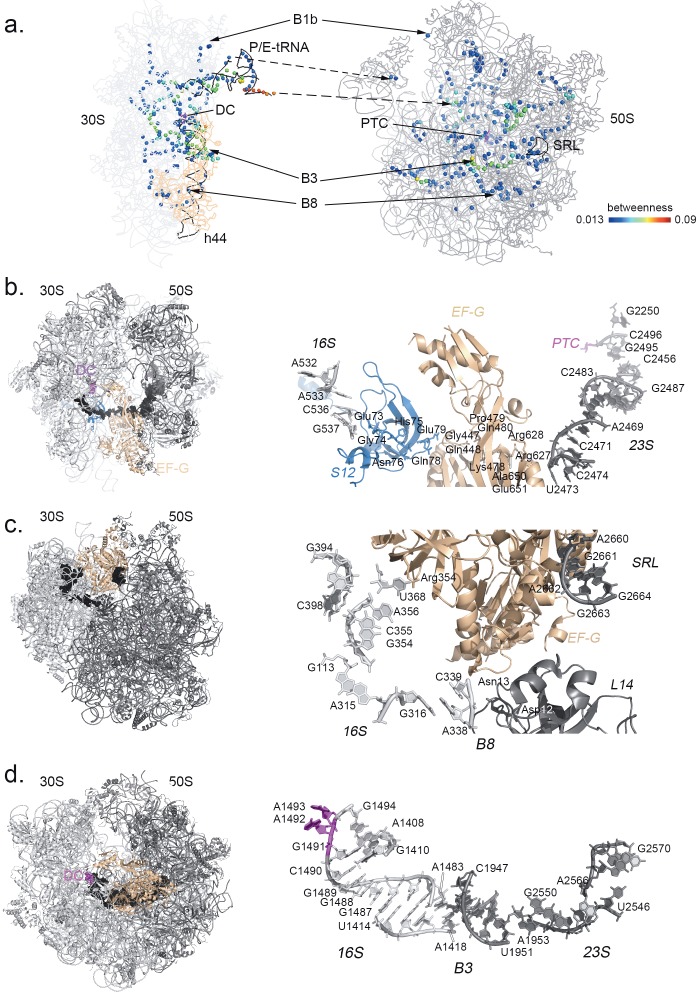
Hub residues with high betweenness values form distinct pathways in the complex at the translocation state.

EF-G promotes the movement of tRNAs and mRNA on the small subunit; however, the exact mechanism of this action is still debated (Yamamoto et al., 2014) . After the translocation, EF-G dissociates from the complex, most probably by the help of GTP hydrolysis on its catalytic site releasing the protein from its docking site on the sarcin-ricin loop (SRL) (Yamamoto et al., 2014) . Here, betweenness of residues on the ribosome in its complex with EF-G at the intermediate state of translocation is investigated as a case study to show the use of this centrality measure in indicating potential allosteric pathways. When the residues at the top 0.05 quantile are displayed on the complex, these residues interestingly reveal pathways linking different sites of the structure both through bonded interactions and nonbonded interactions, such as hydrogen bonding or Watson–Crick pairing (Figure 6a).

From these, three pathways connecting distant functional sites of 30S and 50S are studied; these are the A-site of the small subunit and PTC, docking sites of EF-G on small and large subunits, and DC and PTC.

On the EF-G.70S complex, a string of hub residues is observed to line up between A532 on helix 18 of 16S, which is next to DC residue G530 and C2456 on 23S near PTC (Figure 6b). These two distant sites are linked by residues on ribosomal protein S12 (Glu73, His75, Asn76, Gln78, Glu79), EF-G (Gly447, Gln448, Lys478, Pro479, Gln480 on domain III; Ala626, Arg627, Arg628 on domain V), and 23S (G2455-C2475 on helix 89; G2495 and C2496).

DC residues A1492 and A1493 do not take part in this ensemble. Interestingly, all these regions and residues have critical roles in translation. The highly conserved A532 is located at the A-site on the small subunit and is in direct contact with mRNA, while any disarrangement around the 530 loop region effects the fidelity in translation (Spirin, 1999) . Ribosomal protein S12 has been long known to act as a control element in tRNA recruitment to the complex (Yates, 1979) . A previous site-directed mutagenesis and gene replacement study on the Thermus thermophilus ribosome provided strong evidence of an allosteric communication between DC and EF-Tu linked by S12 and A/T-tRNA (Gregory et al., 2009). The highly conserved QEH triplet of S12 (Gln78, Glu79, His80) was proposed to take a role in transmitting the codon recognition signal to EF-Tu (Gregory et al., 2011). Moreover, the antibiotic dityromycin binds the exact region predicted by our hub residue analysis, as demonstrated by structural studies on Escherichia coli (Bulkley et al., 2014).

Another set of hub residues appears to link Arg354 of EF-G and U368 on helix 15 of 16S to the intersubunit bridge B8 residues, namely C339 on helix 14 and Asp13 on

L14 (Figure 6c). Previously reported ribosomal ambiguity mutations on G347 on helix 14 of B8 suggested that this bridge has an allosteric inhibition effect on the GTPase activity of EF-Tu, even though it is distant from the EF binding site (Fagan et al., 2013). Here, calculations clearly indicate that stability/instability in the B8 region can effect the flexibility of EF-G through helix 14 and helix 15, while impact in the reverse direction is also plausible.

Moreover, flexible SRL residues A2660-G2664 on H95 of 23S rRNA appear as hubs symmetrical to U368, both holding EF-G. The SRL is another important functional region neighboring the active center of EFs. Interactions of the catalytic residue His85 of EF-Tu with nucleotides G2661 and A2662 on the SRL are critical for GTPase activation and hydrolysis (Schmeing et al., 2009; Voorhees et al., 2010) , and it was suggested that the long SRL itself is indispensable for anchoring EF-G to the ribosomal complex and therefore for translocation of mRNA and tRNAs (Shi et al., 2012) . An extensive modeling study on the bacterial ribosome, mapping antibiotic binding motifs based on structural evidence throughout the structure, also highlighted the attractiveness of the SRL for designing new effective drugs (David-Eden et al., 2010).

Figure 6d displays hub residues lining up between DC (A1492, A1493) and near PTC by crossing the intersubunit bridge B3. Similarly, we recently reported the potential allosteric pathways linking DC and PTC, which followed the same trace of residues (Guzel and Kurkcuoglu, 2017). The stable RNA–RNA bridge B3 pivots the ratcheting motion of the subunits, but it may also transfer any information in the form of conformational changes between subunits; therefore, it is an attractive site for drug design. Another important region revealed by the current hub residue analysis covers nucleotides 1408/1409/1491, which confer resistance to several aminoglycosides in Thermus thermophilus (Gregory et al., 2005).

In conclusion, the current approach to describe intermolecular interactions and to construct a residue network of the ribosomal complex succeeds in differentiating residues critical in function or in allosteric communication, among many others. In addition, it strongly agrees with a previous study on ribosomal networks (David-Eden and Mandel-Gutfreund, 2008). The supramolecule ribosome presents an exceptional residue network harboring multiple functional sites highly distant from each other. It astonishingly maintains an effective communication through residues with high betweenness scores, which can transmit perturbations in the form of conformational changes. Moreover, the current findings assemble many distinct observations on allostery employed by the ribosome onto the same map and propose a new model to test how residues can be linked to each other to form pathways for allosteric communication.

Centrality analysis of the different translational states of the ribosomal complex indicates that especially degree and betweenness scores of the same residues change from one conformation to another as the contact topology changes. ehTrefore, as this study suggests, interpretation of findings should be assured by investigating different conformations of the protein complex if possible. Finally, intersubunit bridges B1b, B3, B5, B7, and B8 emerge as attractive sites for drug design to kill drug-resistant bacteria.

## Acknowledgment

This work was supported by the İstanbul Technical University Scientific Research Projects Foundation [Project No: 36110].
